# Past visual experiences weigh in on body size estimation

**DOI:** 10.1038/s41598-017-18418-3

**Published:** 2018-01-09

**Authors:** Joanna Alexi, Dominique Cleary, Kendra Dommisse, Romina Palermo, Nadine Kloth, David Burr, Jason Bell

**Affiliations:** 1 School of Psychological Science, University of Western Australia, Crawley, WA 6009 Australia; 2 ARC Centre of Excellence in Cognition and its Disorders, University of Western Australia, Crawley, WA Australia; 3 Department of Neuroscience, Psychology, Drug Research and Child Health, University of Florence, Firenze, FI Italy; 4 Department of Translational Research on New Technologies in Medicine and Surgery, University of Pisa, Pisa, PI Italy; 5 School of Psychology, University of Sydney, Camperdown, NSW Australia

## Abstract

Body size is a salient marker of physical health, with extremes implicated in various mental and physical health issues. It is therefore important to understand the mechanisms of perception of body size of self and others. We report a novel technique we term the *bodyline*, based on the numberline technique in numerosity studies. One hundred and three young women judged the size of sequentially presented female body images by positioning a marker on a line, delineated with images of extreme sizes. Participants performed this task easily and well, with average standard deviations less than 6% of the total scale. Critically, judgments of size were biased towards the previously viewed body, demonstrating that serial dependencies occur in the judgment of body size. The magnitude of serial dependence was well predicted by a simple Kalman-filter ideal-observer model, suggesting that serial dependence occurs in an optimal, adaptive way to improve performance in size judgments.

## Introduction

The size of a human body is an important marker of physical health^1^. Body Mass Indexes (BMI: mass divided by squared height) of less than 18.5 are clinically underweight^2^, and can be an important severity marker for Anorexia Nervosa^3^; BMIs greater than 30 are diagnostic of obesity^4^. These extremes are linked to a range of poor health conditions, such as cardiovascular disease, Type 2 diabetes and high blood pressure^5^, as well as mental health problems, including low self-esteem^6^ and psychopathologies such as impulse control disorders^7,8^.

Recent research has demonstrated that human observers are often poor at estimating their own body size, and the size of others^9–11^. Crucially, body size judgments are not always veridical, but can be biased by various factors. Perceptions can be biased after a prolonged exposure, or adaptation, to a given body type: viewing thin body shapes for a period of time causes subsequently viewed neutral body shapes to appear larger than they do without adaptation, and the opposite occurs after exposure to large bodies^10,12–15^. Another type of bias is “central tendency”, or “regression to the mean” a well-known phenomenon in magnitude estimation^16^. Cornelissen and colleagues^11^ have shown that healthy females systematically *underestimate* the weight of overweight females, and *overestimate* the weight of thin females, so errors in magnitude consistently default towards the mean or median of the set, consistent with regression to the mean.

Another form of bias that can occur when judging sequences of images is termed serial dependence. This refers to errors in perceptual judgments consistent with assimilation of the characteristics of the previous stimulus with the current stimulus (the opposite effect of adaptation, described above). Serial dependencies have been observed for a range of visual processes, such as in the perception of orientation^17,18^, number^19^, face identity^20^, attractiveness^21,22^ and gender^23^. Although serial dependence results in biasing of perception away from veridical, it has been argued that this is in fact advantageous, increasing efficiency^19,24^, and facilitating the temporal continuity of our perceptual experience^17^.

The primary goal of this study was to test for serial dependence in body size estimation. We did this by using a novel technique for measuring perceived body size, which we term the *bodyline*. It is similar to the numberline technique, now extensively used in studying number perception^25^, in which subjects position a number along an analogue line, mapping number onto space. Here we ask participants to position a body image along a delineated line, mapping perceived size onto space. The technique is simple, intuitive and reliable. We hypothesized that serial dependencies would occur with judgments of body size.

## Results

**Figure 1:**
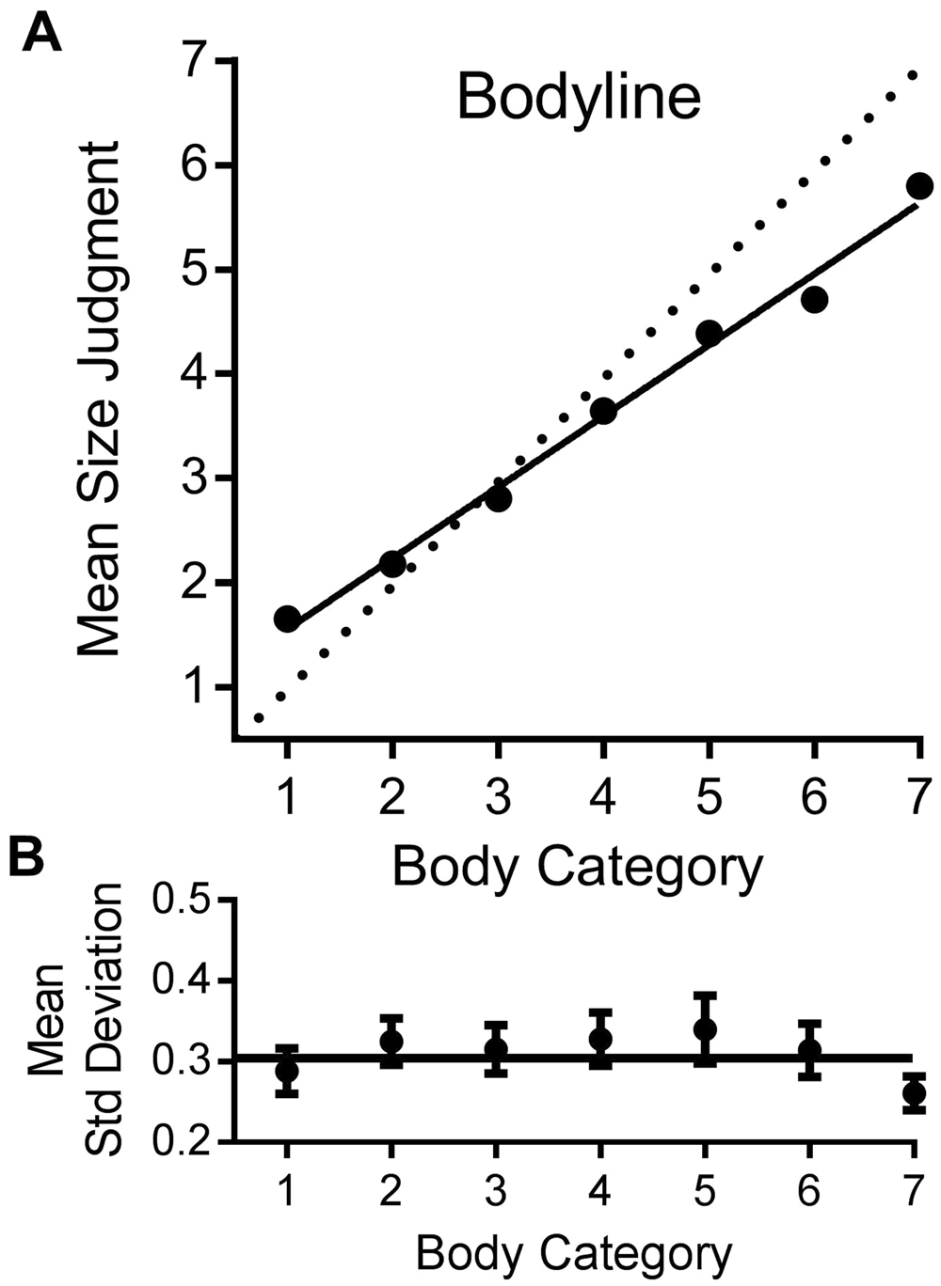
Average performance in the bodyline task. (**A**) Mean size judgments given to each of the seven categories of body, which varied from very thin to very overweight. Error bars represent ± 1 s.e.m. The solid line represents the best fitting linear regression (slope 0.68, R^2^ = 0.99). The dotted line represents linear use of the bodyline, without scaling. (**B**) Average precision thresholds, given by standard deviation of bodyline judgements, as a function of body category. Bars show 95% confidence intervals, almost all of which span the mean, suggesting that precision varied little with body size.

We first verified the efficiency of the method to measure perception of body sizes allocated to different categories. Fig. 1A plots the mean size judgment given to each of the seven body categories, on linear axes. The data show that mean body size judgments increase monotonically, and almost linearly with physical body size (R^2^ = 0.99 for linear fit). This suggests that the size categories were perceived as equidistant. The slope of the linear regression line fitted to the data is less than one, at 0.68 (95% CI: 0.66–0.70). Note that in our study the physical weight of the bodies is not known, and therefore the amount of regression to the mean in our data cannot be precisely calculated. Despite this, our estimated slope is almost identical to that found by Cornelissen and colleagues^11^ (0.72). Those authors used a different technique for estimating body size but inferred strong regression to the mean in body size estimation, based on a slope less than one. Following the conventions of Cicchini, *et al*.^26^ we define a regression index as the difference from unit slope, an index ranging from zero to 1 (where 1 = total regression to the mean). For the averaged data, this is 1 − 0.68 = 0.32 (individual data discussed below).

**Figure 2:**
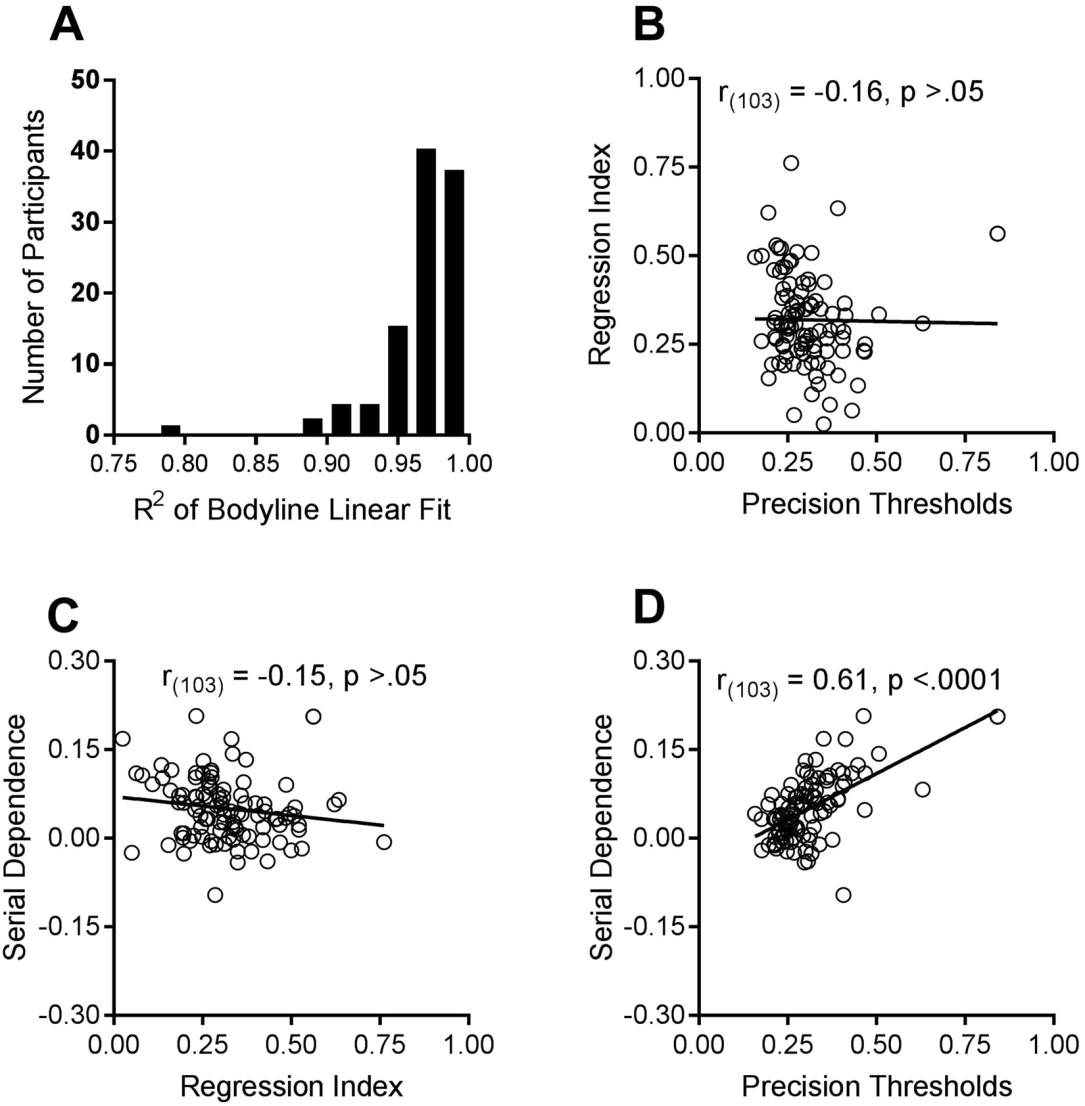
Individual data for the bodyline task. (**A**) Histogram showing the distribution of coefficients of determination (R^2^) for the linear fit. Most are above 0.95, suggesting that the categories were perceived as equidistant, and mapped accurately, save for a scaling constant. (**B**) Regression indexes of the individual subjects (1 minus the slope of best fitting linear regression to their bodyline data) as a function of precision thresholds, defined as average standard deviations for judgments at each category. There is no significant correlation between the two variables. (**C**) Magnitude of serial dependence of individual subjects (defined as the slope of the regression line for similar previous body sizes, illustrated in Fig. 3) as a function of regression index. Again there is no significant correlation, indicating that the two processes are independent. (**D**) Magnitude of serial dependence as a function of precision thresholds. There is a strong and significant correlation, with higher thresholds leading to greater dependency, as predicted by the Kalman filter model (eqn. 8). The top right data point in (**D**) is not an outlier but nevertheless we re-ran the analysis without this individual. The correlation remained highly significant: r_(102)_ = 0.56, p < 0.0001.

Participants reported that they found the bodyline technique intuitive and natural. The individual data suggest that they were very consistent in their judgments. The results of all individual participants were well fit by a linear regression, with R^2^ > 0.95 for the majority of subjects, and R^2^ always greater than 0.78 (see Fig. 2A). Another indication of the consistency of the results is the precision of line-placement. Figure 1B plots the average standard deviations for the bodyline positioning as a function of body category. These are a measure of subject precision, or reliability, calculated separately for each subject, then averaged. There is very little variation in precision with category, except for the extremes, particularly 7, the only point where the 95% confidence intervals do not embrace the global mean. The standard deviations for individual subjects, averaged over categories, are plotted on the abscissa of Fig. 2B. The average standard deviation is 0.35 category units, ±0.02 (95% confidence). The ordinate plots the regression index of the individual subjects. The larger the index, the more participants’ responses have tended towards the mean judgment. Again, there was a good deal of variability (Mean = 0.32, SD = 0.13; 95% CI: 0.29–0.34). However, there was no significant correlation between the regression index and precision, as may be expected on theoretical grounds (see modelling section).

**Figure 3:**
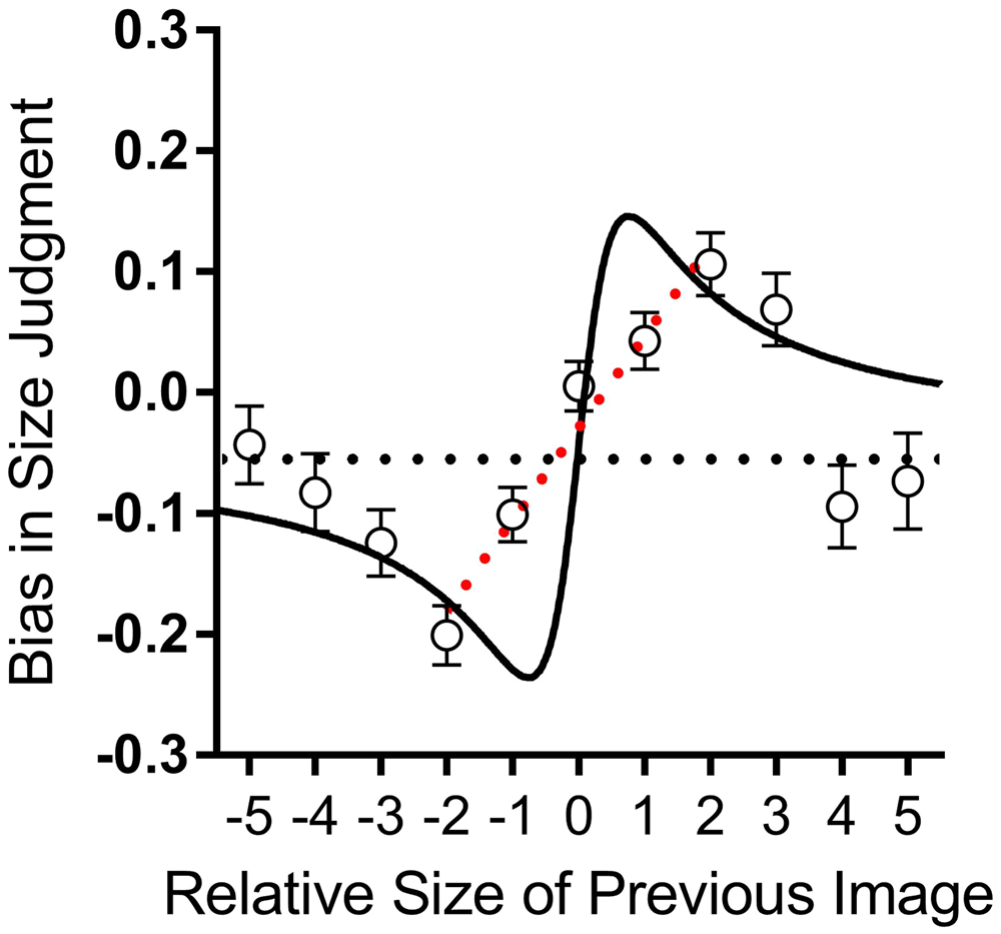
Serial dependencies in body size estimation. Data show the average biases in the perceived size (difference between perceived and physical size), as a function of the difference in size of the body on the preceding trial. Data are averaged over all observers and body categories. Error bars represent ± 1 s.e.m. The continuous curve shows the predictions of the parameter-free Kalman filter model (eqn. 8). The horizontal dotted line plots the average bias, which is slightly negative.

### Serial Dependence in Body Size

The main goal of this study was to test for serial dependence in body size estimation, that is, whether body size judgements were influenced by the preceding trial. Figure 3 plots the average bias in judgements (the difference between the response and the physical stimulus: (*R*_*i*_ − *X*_*i*_ as a function of the difference between the physical sizes of the present and past stimuli (*X*_*i*_ − *X*_*i*−1_). The results were averaged across all participants and all stimulus sizes of the current trial (*X*_*i*_). The data clearly show that body size judgments are systematically biased *towards* prior experience: bodies were perceived as smaller when preceded by a smaller body (lower left quadrant) and perceived as larger when preceded by a larger body (upper right quadrant).

The assimilative bias increased with the difference in size of past and present images, up to a maximum effect for a difference of two body size units, and then reduced in magnitude for larger size differences. This selectivity indicates a highly sophisticated system that assimilates across small but not large size differences. The average data contain a small negative bias (represented by the horizontal dashed line in Fig. 3), which passes through the standard errors of the extreme points. The solid curve which generally followed the pattern of the data in Fig. 3 is the prediction of an ideal-observer model detailed in the next section (eqn. 8). The curve clearly captures the main features of the data: the initial sharp increase in bias, followed by a reduction as the size difference between past and previous stimuli increases. The fit of the model is good, with R^2^ = 0.56, particularly good when considering that there are no free parameters in the model.

We next examined whether there was any relationship between the magnitude of serial dependence and regression to the mean. A robust estimate of the magnitude of serial dependence of each subject was obtained by fitting a linear regression to the five points nearest to the current stimulus, from −2 to +2 units, as illustrated by the dashed line of Fig. 3 for the aggregate subject data. Figure 2C plots this estimate for each subject against their regression index. There was no significant correlation between the two indexes, suggesting they are independent processes.

We also looked for a correlation between the magnitude of serial dependence and precision thresholds of individual subjects. Figure 2D shows there was a strong and significant positive relationship between these two variables, with correlation coefficient *r* = 0.61 (*p* < 0.0001). This relationship is predicted by the Kalman filter model described below. The magnitude of serial dependence bias should depend on their noisiness, with greater dependency over a larger range.

## Modelling

### Central tendency in magnitude estimation

Regression to the mean has recently been formulated in Bayesian terms^26,27^, in which the mean can be considered a *prior*, the a priori “best guess” before any measurement is made. The *prior* is statistically combined with the sensory information, termed the *likelihood*, to yield the most efficient estimate of the judgment (termed the *posterior*). They show that although it leads to systematic biases towards the mean, this strategy can reduce the overall error (which is a combination of bias and precision) by increasing the precision. The *ideal* weight given to the prior (*W*_*P*_) should be proportional to the relative reliabilities (inverse variances) of the *prior* and the *likelihood*.

1 $${w}_{P}=\frac{{\sigma }_{P}^{-2}}{{\sigma }_{P}^{-2}+{\sigma }_{L}^{-2}\,}$$

where *W*_*P*_ is the ideal weighting of the prior, and ${\sigma }_{P}^{-2}$ and ${\sigma }_{L}^{-2}$ are the inverse variances of prior and likelihood respectively. Cicchini, *et al*.^26^ demonstrated that the *optimal prior width* (to minimize error) should depend on both the variance of the likelihood and the range from which stimuli are drawn:

2 $${\sigma }_{P}^{2}=2{\sigma }_{X}^{2}-{\sigma }_{L}^{2}$$

where *σ*_*P*_ is the predicted width (standard deviation) of the prior, *σ*_*X*_ the standard deviation of the range of stimuli and *σ*_*L*_ the standard deviation of the responses. In the current study, the response range was 1–7 (standard deviation 2.1), and standard deviation of the response (likelihood) was on average 0.35 units:

3 $${\sigma }_{P}^{2}=2\times {2.1}^{2}-{0.35}^{2}=8.70$$

Substituting the reliabilities of the prior and likelihoods into equ. 1 shows that the ideal weight given to the prior, for maximum reduction of error, should be 0.014, very low indeed. This weight would change the slope of the responses from 1.0 to 0.977, a regression index of 0.023, far lower than Cornelissen and colleagues^11^ index of 0.28 or our estimated index of 0.32. We must therefore conclude that the measured central tendency in this and previous studies does not represent an optimal encoding strategy.

### Serial dependencies

We model serial dependencies using the Kalman filter model of Cicchini, *et al*.^26^ which assumes that the response *R*_*i*_ to the current stimulus *X*_*i*_ is given by a weighted sum of the current and previous stimuli:

4 $${R}_{i}={w}_{i-1}{X}_{i-1}+(1-{w}_{i-1}){X}_{i}$$

where *w*_*i−1*_ is the weight given to the previous stimulus. Multiplying out and rearranging predicts the response bias, the difference between response and physical body size (*R*_*i*_ − *X*_*i*_) is given by:

5 $${R}_{i}-{X}_{i}={w}_{i-1}({X}_{i}-{X}_{i-1})={w}_{i-1}d$$

where *d* is the difference in physical size between current and previous stimuli (*X*_*i*_ − *X*_*i*−1_). According to Cicchini and colleagues’^26^ model, the weight for the ideal observer is given by:

6 $${w}_{i-1}=\frac{{\sigma }_{i}^{2}}{{\sigma }_{i}^{2}+{\sigma }_{i-1}^{2}+{d}^{2}}$$

Put simply, the weight should increase with the uncertainty of judging the current stimulus (${\sigma }_{i}^{2}$), decrease with the uncertainty of the previous stimulus (${\sigma }_{i-1}^{2}$), and also decrease with the squared distance between the current and previous stimulus. As the average root-variance of subjects’ bodyline judgements did not depend strongly on body category (see Fig. 1B), we used the average, i.e. ${\sigma }_{i}={\sigma }_{i-1}=\sigma$. Eqn. 3 then simplifies to:

7 $${w}_{i-1}=\frac{{\sigma }^{2}}{2{\sigma }^{2}+{d}^{2}}$$

And the predicted bias becomes:

8 $${R}_{i}-{X}_{i}=\frac{{\sigma }^{2}d}{2{\sigma }^{2}+{d}^{2}}=\frac{d}{2+{(d/\sigma )}^{2}}$$

The solid curve which generally followed the pattern of the data presented in Fig. 3 represent the predictions of this parameter-free model, with a fit of R^2^ = 0.56. Inspection of Eqn. 5 shows that the amount of bias should depend on *σ*, the noisiness of subjects’ judgments. The bias should continue to increase with *d* where *d* is <*σ*, so the larger *σ* is, the greater the effects should be, over a larger range. Figure 2D shows that the magnitude of the bias does increase with *σ*, with correlation coefficient *r* = 0.61 (*p* < 0.0001), providing strong support for Burr and Cicchini’s theory^24^.

It has been pointed out that serial dependencies do predict a regression to the mean^19^. This is primarily because categories towards the ends of the scale will tend to be preceded by trials that are closer to the mean than they are, hence draw the response towards the mean. We estimated the expected magnitude of serial dependencies on the average judgements with a simple Monte Carlo simulation, calculating the expected response *R*_*i*_ by reiterating Eqn. 5 for all possible combinations of current and previous stimuli. *w*_*i-1*_ was calculated from Eqn. 7, with σ given by the measured average observer standard deviation. The predicted regression index was 0.014, far less than Cornelissen and colleagues^11^ estimate of 0.28, or our regression index of 0.32. Indeed, the predicted regression index is not too far from that predicted by the ideal observer (0.023), discussed above. This reinforces the suggestion that the two effects are independent of each other, and that regression to the mean does not correspond to an optimal encoding strategy. Instead, the smaller serial dependence could well reflect an optimal encoding strategy.

## Discussion

We devised a novel technique for measuring the perception of body size, which subjects found easy and intuitive. Bodyline judgments were very precise, with average standard deviations of about 0.06 of the scale (0.35 category-units). For all subjects, responses varied linearly with size category, accurate up to a scaling factor.

Despite the linearity and reliability of the judgments, they were not veridical. The data for all subjects was consistent with regression to the mean. While we cannot confirm its magnitude in our data, our estimate was near identical to that reported previously^11^. Regression to the mean is a well-known and ubiquitous perceptual phenomenon^16^ recently explained in Bayesian terms^26,27^ considering the mean as a *prior*. Within the Bayesian framework, Cicchini, *et al*.^26^ calculate the ideal amount of regression to the mean, given the stimulus range and the precision of subjects’ judgments (Eqn. 2). This predicts far less regression to the mean than others have reported^11^, leading us to conclude that the central tendency observed in body size estimation does not represent an optimal encoding strategy. Perhaps it is simply a tendency for subjects not to use the entire available scale, despite the fact that the anchors flanking the scale at all times were clearly beyond each end of the bodyline. This idea is also supported by the fact that there was no significant correlation between the regression index and serial dependence.

On the other hand, the magnitude of serial dependence was well predicted by the ideal Kalman filter model of Cicchini, *et al*.^26^. The reliance of the effect on the difference in size between the current and present stimulus was well predicted, as was the overall magnitude, with a fit of R^2^ > 0.5 for a parameter-free model. Furthermore, the magnitude of serial dependence depended strongly on the precision of each observer, increasing with decreasing precision (increasing thresholds). All this suggests that serial dependencies operate in a flexible, adaptive way to improve performance in size judgments.

Biases in perceived body size owing to visual adaptation have been widely studied^10,12,15,28,29^. Within this literature, clear differences in the magnitude of body size after-effects observed for eating disorder groups compared with healthy controls have been reported. These differences suggest that adaptation processes are disturbed in those suffering from an eating disorder^28^, which might contribute to the body size misperceptions seen in those with an eating disorder. Further light may be shed on the nature of these differences by new research showing that body size is coded within two separable dimensions, body fat and muscle mass^10,30^. However, it remains to be seen whether, and how strongly, serial dependencies contribute to the body-image distortions, observed in those with eating disorders, such as anorexia and obesity^11,29,31,32^, but previous research suggests that they might. For example, it has been reported that variability in body-size judgments is associated with a broad range of eating disorder symptoms, including body-image distortion, binge eating and dietary restraint^32^. Body-image variability then is of clinical relevance. Our results show that serial dependence is strongly correlated with variability in body-size judgments (Fig. 2D), thus suggesting a possible perceptual explanation for these effects. The bodyline task provides a simple yet sensitive method of investigating whether the newly discovered serial dependencies in perception contribute to eating disorder symptoms and to specific body-image distortions observed by those at the extremes of the BMI continuum. The method could also prove useful in determining the circumstances under which adaptation rather than serial dependencies occur, since these are far from clear^17,24^.

While the current research contributes important new information about serial dependencies and body size perception, a few considerations should be acknowledged. First, since the precise body weights of the female stimuli in our study are not known, we could not accurately measure the overall magnitude of regression to the mean in body size estimation in our data. Although our data resembles what would be expected from regression to the mean, an accurate estimate would require one to use stimuli for which body weight is known. Note however that in research that met this criterion^11^, an almost identical estimate of regression to the mean was reported (slope of 0.72 vs. our 0.68). Additionally, while this study did not assess male participants on this task, or use male stimuli, it seems likely that a similar pattern of results would be obtained under those circumstances. Thus far, serial dependencies have been shown to occur across a wide range of visual processing tasks^17,20–22,24^, suggesting they commonly occur in visual perception. We note that misperceptions in the male assessment of ‘muscular ideals’ have been observed^33^ but it remains to be seen if serial dependencies contribute to these biases. Finally, the current study recruited undergraduate university students, which may have influenced the generalizability of the findings. It is important to extend the current research to a general community sample, where one can expect greater variability in age and BMI, and we intend to do so.

The current research developed a novel technique for examining biases in perceived body size. Our data are consistent with previous reports of regression to the mean in body size judgments^11^. Additionally, our method reveals evidence of a serial bias in perceived body size, with size judgments tending towards that of the previously viewed body. These findings add to the growing body of literature describing the range of visual processes for which serial dependencies occur.

## Method

### Participants

Following standard practices in body size perception research, we restricted our subject sample and stimuli to females. Written informed consent was obtained from all participants. We recruited 103 young female undergraduate psychology students from The University of Western Australia, ranging in age from 17 to 25 years (*M* = 18.88, *SD* = 1.65). This sample size was chosen in order to obtain a robust measure of serial dependence. BMIs in this group ranged from 16.23 to 43.99 (*M* = 22.22, *SD* = 3.93). Three additional participants were excluded from the study. Two participants clearly did not attempt to follow instructions to complete the experiment (they had clicked the same area on the *bodyline* throughout the task). The third participant was excluded because of a computer malfunction. Participants received partial course credit for completing the study. The experimental procedure was approved by the University of Western Australia’s Human Research Ethics Committee and the experiment was performed in accordance with their guidelines and regulations.

### Materials and Procedure

The experiment was performed on a host Asus PC running Matlab^34^ and the Psychophysics Toolbox^35^. Stimuli were displayed on a ViewPixx monitor with a resolution of 1980 × 1024, showing stimuli at 120 Hz. Each pixel subtended 1′ of visual angle at a set viewing distance of approximately 870 mm and the mean luminance of the display was 50.4 cd/m^2^. Data were analysed using GraphPad Prism software.

### Stimuli

An initial set of 71 colour images of female bodies (9.6 cms × 9.6 cms), clothed in either swimwear or underwear, were sourced from the internet and printed onto cards. They were selected to range from underweight through normal-weight to overweight and obese. Images were cropped in Adobe Photoshop to display the whole body but omit the face, to ensure that facial attractiveness did not bias participants or distract from the judgement of body size. An initial pilot study involved 16 raters (chosen to be broadly comparable to those in the main study) who were asked to group these images into seven distinct categories, ranging from thinnest to largest, along the body size and weight continua. Participants were presented with the stack of body image cards, in no particular order and were instructed to place each of the body images into one of seven categories. Participants were permitted to reconsider their choices during the task. Images were then ranked according to inter-rater agreement and the best category exemplars (those with a clear mode) were chosen for each category, leaving 35 body images in total (five images per category).

In the bodyline experiment, the 35 female body images (approximately 6.5° × 6.5°) were presented on the ViewPixx display for 250 ms each. To reduce visible persistence and colour after images the RGB values in the image were compressed by 80%, towards the middle of the range. Immediately after the stimulus, a large high-contrast visual noise mask (measuring 11° × 11°), comprised of scrambled versions of all the body images, was presented to minimise any visible persistence of the body image.

### Procedure

**Figure 4:**
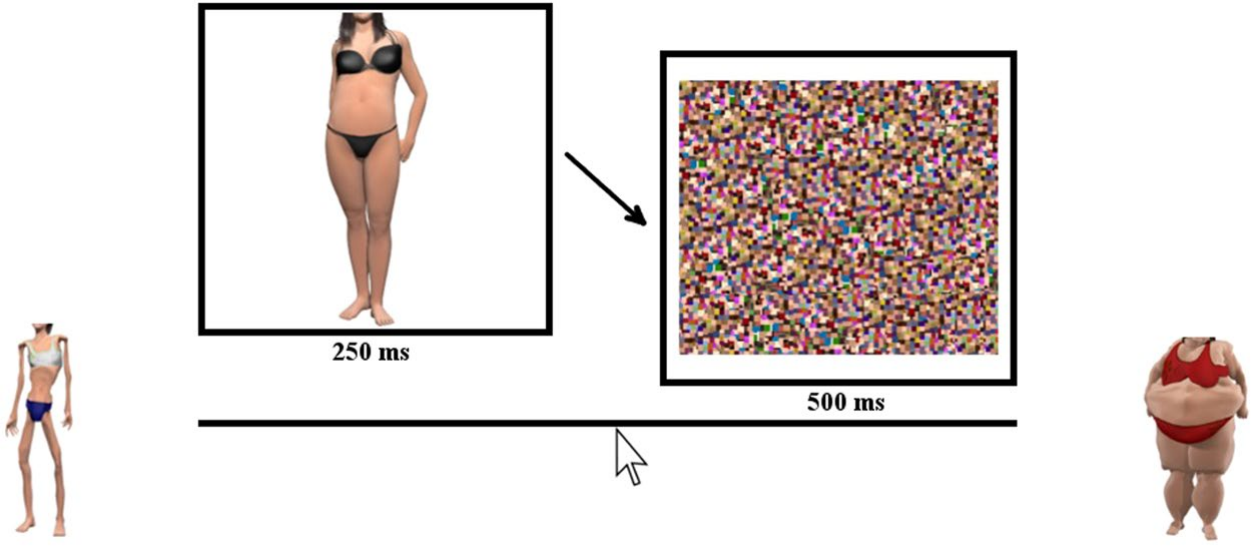
Visual depiction of the bodyline task, in which a female body image was presented for 250 ms, immediately followed by a visual noise mask for 500 ms. Participants indicated the perceived size of the image by clicking on the bodyline delineated with extreme female bodies as anchors presented a further unit of scale beyond the bounds of the numberline. For illustration purposes the females are represented by synthetic body images created in Poser®^36^.

Participants were seated in a quiet room facing a computer screen and keyboard. They were required to judge the perceived size of a briefly presented body stimulus by positioning a marker along the *bodyline*, a visual analogue scale, consisting of an unmarked line scored linearly as 1.0–7.0. The scale was displayed throughout the experiment, together with anchor body images offset one additional unit (1/7^th^ of the scale) from each end of the bodyline, severely underweight on the left, and severely overweight on the right (see Fig. 4). Prior to commencing the study, participants were informed that these anchors were more extreme than any of the body images to be shown in the experiment. On each trial a body was presented for 250 ms, followed immediately by the visual mask for 500 ms. Responses were made by left-clicking the mouse button along the bodyline.

Participants completed 14 practice trials, followed by 3 blocks of 50 experimental trials. The practice trials encompassed the entire range of images, two samples from each category. Participants were then told that this was the entire range, and reminded that the anchor images were more extreme than the bodies to be judged. Images from each of the seven categories were presented in a fixed order across all subjects. Our experimental sequence ensured that in each run of 50 trials, each body size category both preceded and followed each other category, including its own. Lastly, participants’ height and weight were measured to obtain an estimate of Body Mass Index (BMI).

### Data Availability

The datasets created and analysed during the current research are available from the corresponding author on reasonable request.
